# Utility of a Dengue-Derived Monoclonal Antibody to Enhance Zika Infection In Vitro

**DOI:** 10.1371/currents.outbreaks.4ab8bc87c945eb41cd8a49e127082620

**Published:** 2016-07-05

**Authors:** Anu Susan Charles, Rebecca C. Christofferson

**Affiliations:** Pathobiological Sciences, Louisiana State University, Baton Rouge, Louisiana, USA; Pathobiological Sciences, Louisiana State University, Baton Rouge, Louisiana, USA

**Keywords:** antibody, dengue, enhancement, Zika, zika viru

## Abstract

Introduction: Zika virus (ZIKV) has emerged in dengue (DENV) endemic areas, where these two related flaviviruses continue to co-circulate. DENV is a complex of four serotypes and infections can progress to severe disease. It is thought that this is mediated by antibody dependent enhancement (ADE) whereby antibodies from a primary DENV infection are incapable of neutralizing heterologous DENV infections with another serotype. ADE has been demonstrated among other members of the Flavivirus group.

Methods: We utilize an in vitro ADE assay developed for DENV to determine whether ZIKV is enhanced by a commonly available DENV serotype 2-derived monoclonal antibody (4G2).

Results: We show that ZIKV infection in vitro is enhanced in the presence of the 4G2 mAb.

Discussion: Our results demonstrate that ADE between ZIKV and DENV is possible and that the 4G2 antibody is a useful tool for the effects of pre-existing anti-DENV antibodies during ZIKV infections.

## Introduction

Flaviviruses are positive strand RNA viruses of medical and veterinary significance, including dengue virus (DENV), where 390 million cases occur annually, and Zika virus (ZIKV), which has recently emerged in the Americas.^1^^,^ 2 These two flaviviruses are currently co-circulating in much of the tropics, especially in Central and South America.^2^

ZIKV was first isolated in 1947 from a Rhesus macaque from the Zika forest in Uganda^3^^,^ 4 and the first human isolation was recorded in 1954 in Nigeria.^5^^,^ 6 The pathogen since has expanded from the African continent to countries in Asia and to many islands in Pacific Ocean, before finally it emerged in the western hemisphere in 2013.^6^^,^ 7 ^,^ 8 ^,^ 9 ^,^ 10 ^,^ 11 Both DENV and ZIKV human infections are similar in pathognomonic symptoms^12^ and are transmitted by the bite of infected mosquitoes of *Aedes* species, specifically *Ae. aegypti* and *Ae. albopictus.* 13

The four serotypes of DENV1-4 share 60-70% genetic homology and cause either asymptomatic or subclinical infections, mild illness known as dengue fever (DF), or severe manifestations (dengue hemorrhagic fever, DHF, or dengue shock syndrome, DSS).It is thought that the severe forms of DENV are mediated by cross-reactive but not cross-protective immunity to the primary DENV infection whereby antibodies produced in that first infection fail to neutralize a second, heterologous infection with another serotype. This phenomenon, antibody dependent enhancement (ADE), has confounded vaccine development, as the antibody response is often not equitable.^14^ Briefly, ADE is an immunopathological phenomenon in which a non-neutralizing antibody binds DENV and aids entry into cells bearing the Fc-receptor, such as monocytes/macrophages and dendritic cells. This leads to an increase in viral replication and ultimately, increased viral load of the host.^15^ A striking example of ADE occurs in babies born to DENV-immune mothers, upon their first infection with DENV develop a severe form of the disease due to the presence of maternal antibodies.^16^^,^ 17 In addition, there are other reports of enhancement capacity among other flavivirus combinations in both human samples and small animal models. 18 ^,^ 19

Antibody dependent enhancement by Zika infection has not yet been reported in humans, but has been shown in vitro in cell culture.^20^ Herein we investigate whether a dengue-derived monoclonal antibody (4G2) is capable of enhancing ZIKV infection in vitro, providing information regarding this widely available tool for future studies of the immunological interaction among DENV1-4 and ZIKV.

## Materials & Methods

*Virus and Cells*

Virus was generously provided by Dr. Robert Tesh at University of Texas, Medical Branch. ZIKV MR799 and DENV2 16803 were utilized for all assays and a multiplicity of infection (MOI) was determined prior to infection assay. THP-1 cells were provided to us by Dr. Juan Martinez at Louisiana State University. Cells have previously been shown to have low infectivity for DENV2, except in the presence of antibodies, including 4G2.^20^ Vero cells were cultured using M199E (Sigma-Aldrich) and under standard tissue culture conditions (37°C, 5% CO2), with 2% anti-microbial/anti-mycotic and 10% fetal bovine serum. THP-1 cell culture was conducted as in 20 and were also incubated at 37°C and 5% CO2. Both viruses were grown in Vero cells prior to infection of THP-1 cells under standard conditions as above.

*In vitro* *Antibody Dependent Enhancement Assay*

The assay was performed using the protocol in Diamond, et al. with some modifications.^20^ First, we used a multiplicity of infection (MOI) for ZIKV of 2 and a MOI of 10 for DENV2 to account for the difference in infection kinetics of these two viruses^21^. The THP-1 cells (2.5 x 10^5^) were infected with DENV2 or ZIKV by incubating at 37°C for 90 minutes. For antibody treatment, infection was carried out in the presence of virus-antibody complex formed by incubating virus (DENV2 or ZIKV) with approximately 200ng/0.2μL of mAb 4G2 (Anti-Flavivirus group antigen antibody, EMD Millipore) at 37°C for 30 minutes. After infection, cells were washed six times by centrifugation at a speed of 900 x g for 3 minutes. The pellet was finally resuspended in complete medium containing approximately 200ng/0.2μL of mAb 4G2 and was incubated at 37°C for 72 hours before it was centrifuged to separate supernatant and pellet. There were 10 replicates for controls and antibody treatments.

*Virus Quantification*

Viral titer was calculated from the plaque assay on Vero cells whereby 100uL of inoculum at serial dilutions of 1:10 to 1:1000 was pitted onto confluent Vero monolayers in 6-well plates (Corning, Corning, NY) and allowed to incubate on a rocker for 30 minutes. The first overlay of media and low melting agarose immediately followed and plates were placed in the incubator at 37°C at 5% CO2. The second overlay, which included neutral red stain for visualization of plaques, was administered on day 3 post inoculation for ZIKV and day 6 post inoculation for DENV2.

For DENV2 and ZIKV virus-only controls, as well as the DENV2 and ZIKV treatment pellet groups, we used the undiluted samples. Because there were too many plaques to count in the undiluted samples and 1:10 samples of supernatant from the ZIKV treatment groups, we utilized the counts in the 1:100 dilution. Likewise, there were too many plaques to count in most of the undiluted DENV2 supernatant samples, thus we utilized the 1:10 samples.

*Statistical Analysis*

Differences in pfu/ml between treatment groups and controls were analyzed using Student’s T-test in R version 3.2.5. Statistical significance was assessed at the α=0.05 level.

## Results & Discussion

When determining the level of enhancement DENV2 strain 16803 achieved in the presence of 4G2, we found significant differences between titers from the treatment group with the antibody and the control group without the antibody (Table 1). In both the supernatant and pellet, the control group produced little or no plaques while the treatment group had countable plaques, which resulted in a mean value significant from the control group (p-values <0.05). Mean viral titers in the supernatant and pellet, respectively, increased more than 140-fold and 110-fold when pre-treated with antibody.

**Table 1:** Average DENV2 titers calculated based on n=10 replicates from plaque assays. Also reported is the 95% lower confidence limit (LCL) and upper confidence limit (UCL) in the control group (no antibody) and treatment group (with 4G2 antibody).

Virus	Location	Antibody	Mean	95% LCL	95% UCL
DENV2	Supernatant	Yes	1.27x103	1.03x103	1.51x103
DENV2	Supernatant	No	9	-1.14x101	2.9x101
DENV2	Pellet	Yes	1.10x102	2.44x102	1.96x102
DENV2	Pellet	No	0	0	0

Similarly, we demonstrated that ZIKV infection could be enhanced, with significant difference in the viral titers when infection of THP-1 cells was done in the presence of mAb 4G2 compared to controls without mAb 4G2 in both supernatant and pellet (p-values <0.05) as shown in Table 2. ADE by mAb 4G2 resulted in increase in viral titer by more than 60 times in supernatant and 248 times in the cell pellet when compared to its respective controls. The high viral titer in the 4G2 supernatant compared to all other groups could be attributed to the cellular lysis during viral replication, as peak ZIKV replication is often observed the day after supernatant collection in Vero cells, indicating that perhaps all of the virus was cell-free as the majority of THP-1 cells had already been infected and lysed at that point. Figure 1 shows the cytopathic effects on Vero cells following inoculation with either DENV2 or ZIKV treatments. Thus, we have demonstrated that there was substantial enhancement in infection of FcR-bearing THP-1 cells in the presence of the commercially available, monoclonal antibody 4G2.

**Table 2:** Average ZIKV titers calculated based on n=10 replicates from plaque assays. Also reported is the 95% lower confidence limit (LCL) and upper confidence limit (UCL) in the control group (no antibody) and treatment group (with 4G2 antibody).

Virus	Location	Antibody	Mean	95% LCL	95% UCL
ZIKV	Supernatant	Yes	6.00x10^3^	4.74x10^3^	7.26x10^3^
ZIKV	Supernatant	No	9.40x10^1^	4.84x10^1^	1.40x10^2^
ZIKV	Pellet	Yes	2.48x10^2^	5.74x10^1^	4.39x10^2^
ZIKV	Pellet	No	1.00	-1.26	3.26

**A) Vero cell monolayer compared to observed ctyopathic effect from undiluted supernatants of ADE assay in the presence of 4G2 antibody and B) Zika virus or C) DENV2.**
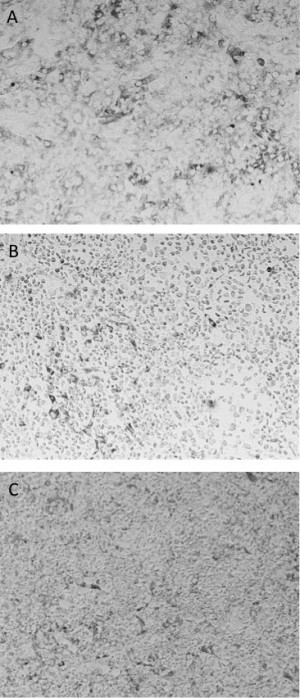

While 4G2 is DENV2-derived, it is broadly used as an anti-flavivirus monoclonal antibody^22^^,^ 23. Thus, it cannot be ruled out that ZIKV could be enhanced by other closely related flaviviruses such as Japanese Encephalitis or Yellow Fever. As the scientific community pushes to fill the gaps in our ZIKV knowledge base, elucidating the potential for interaction with DENV is critical. Understanding the methods and tools available for such investigations is important to move the field of ZIKV research forward. Hence, our results demonstrate not only the utility of a widely available tool for ZIKV research, but also provides further insight into the potential for a role of pre-existing Flavivirus antibody in ZIKV pathogenesis.

## Competing Interests

The authors have declared that no competing interests exist.

## Data Availability

All data is available in Appendix 1.

## Appendix 1

Data from Individual Replicates: Data_Appendix1
